# A systematic review of the quality and scope of decision modelling studies in child oral health research

**DOI:** 10.1186/s12903-021-01680-3

**Published:** 2021-06-25

**Authors:** Greig D. Taylor, Katherine Carr, Helen J. Rogers, Chris R. Vernazza

**Affiliations:** 1 School of Dental Sciences, Faculty of Medical Sciences, Newcastle University, Newcastle upon Tyne, UK; 2 Newcastle upon Tyne Hospital NHS Foundation Trust, Newcastle upon Tyne, UK

**Keywords:** Health economics, Children, Decision analytical modelling

## Abstract

**Background:**

Decision analytic models are often used in economic evaluations to estimate long-term costs and effects of treatment which span beyond the time-frame of a clinical trial, therefore providing a better understanding of the long-term implications of decisions that conventional trial-based economic evaluations fail to provide. This is particularly relevant for considering oral health interventions in children as treatments may affect adult oral health. However, in the field of child oral health there has not been an evaluation of the quality and scope of decision analytical models which extend into adulthood. The aim of this review is to examine the scope and quality of decision modelling studies, with horizons extending into adulthood, within the field of child oral health.

**Methods:**

The following databases were searched: NHS Economic Evaluation Database (CRD York), MEDLINE, EMBASE, CINAHL, Web of Science, Scopus, the Cochrane Library and Econlit. Full economic evaluations, in the field of child oral health, published after 1997 which included a decision model with a horizon that extended beyond the age of 18 years old were included. Included studies were appraised against the Drummond checklist and the Consolidated Health Economic Evaluation Reporting Standards by calibrated reviewers.

**Results:**

Four hundred studies were identified, of which nine met the inclusion criteria. Of the nine, eight were cost-effectiveness models. The majority focussed on the prevention or management of dental caries. The mean percentage of applicable Drummond checklist criteria met by the studies in this review was 82% (median = 85%, range = 54–100%). Discounting of costs and performing an incremental analysis were noted as key methodological weaknesses. The mean percentage of applicable CHEERS criteria met by each study was 82% (median = 87%, range = 32–96%). Justifying the type of model, analytical methods used, and sources of funding were most commonly unreported.

**Conclusions:**

There is a paucity of decision analytical models in the field of child oral health. Most of those that are available are of high methodological and reporting quality.

**Supplementary Information:**

The online version contains supplementary material available at 10.1186/s12903-021-01680-3.

## Background

Dental disease is prevalent and carries a high burden amongst children worldwide. Untreated dental caries [1] and molar-incisor hypomineralisation (MIH) [2] are the most common oral disease in children, with an estimate of 9% [3] and 13.1% [2] respectively of children presenting with these diseases globally. Dental disease may directly impact a child as they could experience pain [4–8], either in isolation, or manifest during sleeping, eating/drinking or carrying out daily activities [6, 9, 10], or have aesthetics and societal implications [11, 12]. Similarly, indirect impacts such as problems with school attendance [13], disturbances to general growth and development [10] as well as placing a burden on the parents, siblings and the wider general society have been reported [6, 14]. It is therefore important, given these direct and indirect impacts, to know how best to manage these dental conditions in children. There is a need for better evidence to synthesise how best to decide to treat these conditions in addition to considering how such decisions made in childhood will impact the patient over their lifetime [15].

For any given dental disease, a range of interventions can be offered to manage the problem and reduce the impact the disease has on the child. Often different treatment options have different costs and effects associated with them and choosing which to provide can be determined by efficiency (i.e. which intervention offers the most benefit for the least cost or the most favourable cost:benefit ratio). This can be assessed using an economic evaluation, the results of which can support decision makers to maximising benefits from limited healthcare resources [16, 17]. Economic evaluations can be run alongside a clinical trial [18]. However, clinical trials have a finite time span (follow up period) and may only compare two mutually exclusive treatment options, where in reality, several may exist. In addition, trial-based economic evaluations use individual patient data, meaning that valid evidence from other trials or meta-analyses may be ignored when a judgement on the most efficient option needs to be made. Therefore using data gathered from a trial is unlikely to capture long-term costs and benefits of alternative interventions [16]. Decision analytic models (DAM) offer a way to model for long-term costs and benefits and can also synthesize evidence from similar trials to improve the robustness of the evaluation. This is hugely beneficial in decisions involving a child’s care as it helps establish the most efficient option over the lifetime of the individual [19].

Previous systematic reviews of economic evaluations in dentistry have either focused on specific areas of dental disease [20–23] or economic evaluation methodologies [24, 25]. For example, Qu et al. [24], reported the methodological quality of DAMs studies involving interventions to manage dental caries in children and adults. However, the scope of this review was only specific to dental caries interventions, included adult patients and did not appraise the quality of reporting [24]. Rogers et al. [26] was the first systematic review to evaluate the scope and quality of economic evaluations in child oral health but excluded modelling studies that extended into adulthood. Excluding these studies meant they could focus on the benefits gained during childhood. However, the problem with this approach was that it precluded analysis of studies assessing interventions with benefits into adulthood. The aim of this study is to report a systematic review, considering both the scope and quality, of decision modelling studies within the field of child oral health which extend the time horizon beyond the age of 18.

## Methods

### Protocol and registration

The study protocol was registered with the Prospective Register of Systematic Reviews (PROSPERO—CRD42020166717), and it followed the recommendations of the Preferred Reporting Items for Systematic Reviews and Meta-Analysis (PRISMA) statement for reporting.

### Search strategy

A search strategy was developed (attached in Additional file 1). The included search terms related to the key concepts associated with the review question. These terms were combined and adapted to comply with the validated NHS CRD economic evaluation filters for MEDLINE, EMBASE and CINAHL. These were adapted, as required for the purposes of this search, for use in the remaining databases which are noted below.

On the 1st October 2020, a search of the published literature was undertaken, by one reviewer (HJR) in the following electronic databases: NHS Economic Evaluation Database (CRD York), MEDLINE, EMBASE, CINAHL, Web of Science, Scopus, the Cochrane Library and Econlit. Searches covered the period from commencement of each database system until the 1st October 2020.

The reference lists of included articles were examined to assess if additional relevant studies, that were not found during the database searches, should be included. Efforts were made to identify relevant unpublished ‘grey’ literature and conference proceedings through appropriate websites and databases such as OpenGrey. The EThOS database was also searched to identify relevant published UK theses.

### Eligibility criteria

For studies to be included in this review, they had to meet the following inclusion criteria:

- Full economic evaluation in the field of child oral health
- Involved a decision model with a horizon that extended beyond the age of 18 years old
- Published after 1997

No language restrictions were placed. It should be noted that cost-minimisation studies may not be universally considered as full economic evaluations, however, it was agreed by the research team to include them in this review. Similarly, it was agreed by the authors that only studies published after 1997 should be included in this review. This was due to the Drummond checklist [27], which was to be employed for this current review, only being available to researchers after this year.

Studies which used the lifetime of the tooth as a horizon were excluded. The reason for excluding these studies is that choosing such a horizon would not reflect the key differences in costs and consequences between interventions, e.g. if an evaluation compares filling and extraction of teeth, and the lifetime of the tooth was chosen as the horizon, once the tooth has been extracted the evaluation would cease yet there will still be benefits from the filling option that extend beyond this. Therefore, in these evaluations, comparing benefits obtained during childhood and adulthood from interventions carried in children cannot be fully assessed.

### Study selection and data extraction

Search results were organised using Endnote™ X9. Duplicate articles were removed. Title and abstract screening against the eligibility criteria, was carried out independently by two reviewers (GDT & KC), with any disagreement resolved by consensus. If necessary, any unresolved differences were resolved by a third reviewer (CRV).

Full texts were obtained for all titles that met these criteria. Two reviewers (GDT & KC) assessed the full texts against the inclusion/exclusion criteria independently, with any disagreement resolved by consensus. If necessary, any unresolved differences were resolved by a third reviewer (CRV).

Two reviewers (GDT & HJR) extracted data and assessed both the methodological quality, independently. A calibration exercise (using 3 papers) was conducted with all reviewers prior to commencement of data extraction and methodological/reporting quality appraisal. Any disagreement was resolved by consensus, and where needed, any unresolved differences were resolved by a third reviewer (KC).

For each selected article, the following data, split into three sections, were extracted:

- *Publication details* publication year; author; country
- *Study Characteristics* oral health condition studied; nature of intervention
- *Economic evaluation characteristics* type of economic evaluation; perspective of the model; costing data sources; data source (primary/secondary data); horizon; model design; model input and parameter details; model outcome

### Quality assessment

An assessment of methodological quality and reporting quality was undertaken. The most widely used checklist, the Drummond 10 item, 13 criteria checklist [27] (a simplified version of the 35-item Drummond version) was used to assess methodological quality. This checklist is recommended in the Cochrane Handbook for Systematic Reviews of Interventions [28].

To assess reporting quality, the Consolidated Health Economic Evaluation Reporting Standards (CHEERS) 24-item checklist was used [29]. Published in 2013, the CHEERS checklist aims to ensure consistent and transparent reporting of economic evaluations is carried out. The simplified Drummond, and CHEERS checklists have been previously used in published systematic reviews of economic evaluations assessing oral health interventions [25, 26]. A score of 0, 1, 2 was allocated for each criterion on the checklist:

- **Score 0:** Criterion not met.
- **Score 1:** Criterion met.
- **Score 2:** Criterion not applicable.

Rogers et al. [26] reported cut-offs in their recent systematic review that appraised the quality and scope of economic evaluations in child oral health up to the age of 18 years old only. High, moderate and low cut-offs were assigned to each study based on their percentage applicability of Drummond [27] and CHEERS [29] criteria. The values used for these cut-offs were:

- Drummond Criteria: High > 50%; Moderate 32–50%; Low < 32% [26]
- CHEERS criteria: High > 83%; Moderate 63–83%; Low < 63% [26]

It was agreed by the research team to use the same cut-offs for this review to allow a meaningful and direct comparison between the two studies.

### Statistical analysis

Simple descriptive statistics, including inter- and intra-rater reliability scores, were conducted on the extracted data and quality appraisal results using IBM® SPSS® Statistics v25. Meta-analyses were inappropriate given the diversity of interventions covered in addition to the aim of the review being to identify good practice and appraise quality.

### Rater reliability

Cohen’s kappa (κ) was calculated at 0.75 (93.2% agreement) for overall inter-rater agreement for the Drummond checklist, and at 0.77 (91% agreement) for the CHEERS checklist. These figures suggest substantial strength of agreement [30]. To determine intra-rater reliability, one of the included studies was randomly selected using an online random number generator and re-reviewed four weeks later. Intra-rater agreement was 1.00 (100% agreement) for both GT and HJR, when applying the Drummond checklist, and 0.71 (91.6% agreement) for GT and 0.84 (95.8% agreement) for HJR when using the CHEERS checklist.

## Results

**Fig. 1:**
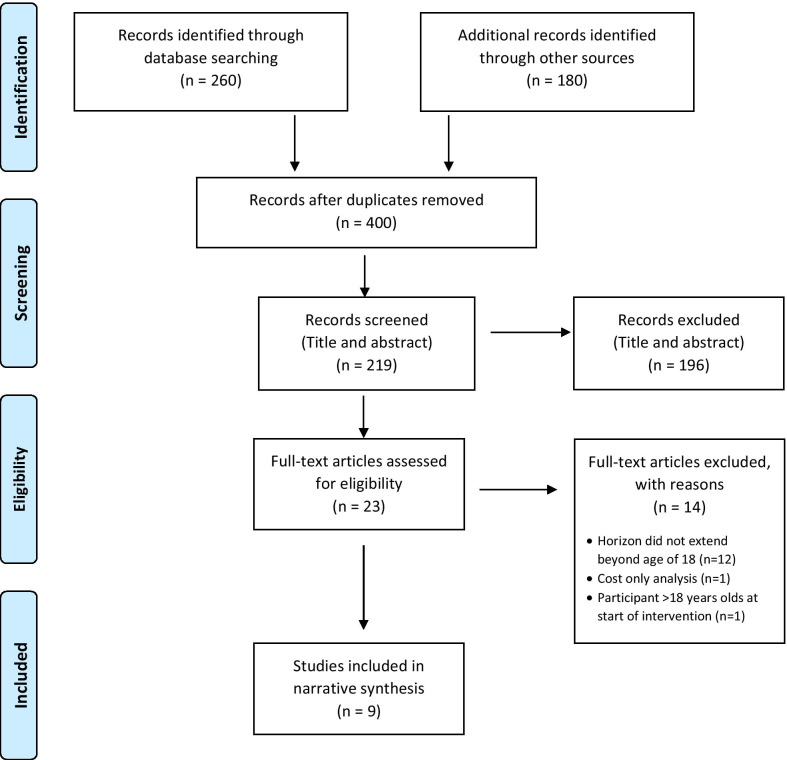
PRISMA: flow diagram of the study

The search resulted in 440 articles and after the removal of duplicates, 400 articles were identified for title and abstract screening. Twenty-three papers were included for review of the full text. Fourteen were excluded because they did not meet the inclusion criteria. Nine articles were included and underwent data extraction. Reference lists of included articles did not produce any further articles for inclusion. A summary of article selection is presented as a flowchart, based on PRISMA guidelines (Fig. 1). The publication and study characteristics and economic evaluation characteristics of the nine included studies are shown in Tables 1 and 2 respectively.

**Table 1:** Publication and study characteristics of each included economic evaluation

Author	Title	Year of publication	Country	Oral health condition studied	Nature of the intervention
Broden et al. [1]	Cost-effectiveness of pulp capping and root canal treatment of young permanent teeth	2019	Sweden	Pulp exposures due to caries	Pulp-capping and root canal treatment in posterior permanent vital teeth
Elhennawy et al. [2]	Managing molars with severe molar-incisor hypomineralization: a cost effective analysis in a German healthcare setting	2017	Germany	Molar-incisor hypomineralisation	Removal and orthodontic alignment of the second and third molar; restore using resin composite; restore using an indirect restoration
Schwendicke et al. [3]	Cost-effectiveness of one- and two-step incomplete and complete excavations	2013	Germany	Caries	One- or two-step incomplete caries removal; complete caries removal
Schwendicke et al. [4]	Detection and treatment of proximal carious lesions: milieu-specific cost-effectiveness analysis	2015	Germany	Caries	Two detection strategies and three treatment options: non-invasive, microinvasive (resin infiltration); invasive (2-surface composite)
Schwendicke et al. [5]	Detecting and treating occlusal carious lesions: a cost effectiveness analysis	2015	Germany	Caries	Four detection strategies and three treatment options: non-invasive, microinvasive (sealing); invasive (1-surface composite)
Schwendicke et al. [6]	Effects of taxing sugar-sweetened beverages on caries and treatment costs	2016	Germany	Caries	20% reduction on tax relating to sugar-sweetened beverage consumption
Schwendicke et al. [7]	In-office application of fluoride gel or varnish: cost-effectiveness and expected value of information analysis	2017	Germany	Caries	No application of fluoride varnish; professional application of fluoride varnish; professional application of fluoride gel
Schwendicke et al. [8]	Cost-effectiveness of caries-preventive fluoride varnish applications in clinical settings amongst patients of low, moderate and high caries risk	2018	Germany	Caries	Fluoride varnish; no fluoride varnish
Splieth et al. [9]	Modelling lifelong costs of caries with and without fluoride use	2008	Germany	Caries	Fluoride exposure

**Table 2:** Characteristics of each included Economic Evaluation

Author	Type of economic evaluation	Model design	Perspective	Horizon	Costing data details	Model input and parameter details	Data sources	Outcome measure
Broden et al. [1]	CEA	Cohort simulation Markov	Care provider	9 years	Swedish Board for Dental Benefits	Systematic review; Swedish Social Insurance Agency during 2009, with a follow-up of 5; Statistics Sweden (mortality)	Secondary data	Extracted tooth or death of patient
Elhennawy et al. [2]	CEA	Individual patient-level microsimulation Markov	Mixed public–private-payer	Lifetime	German public and private dental fee catalogues (BEMA and GOZ)	Combination of systematic reviews and primary studies	Primary & secondary data	Tooth retention years
Schwendicke et al. [3]	CEA	Individual patient-level microsimulation Markov	Mixed public–private-payer	63.5 years	Fee catalogues for the statutory public insurance & private dental catalogue GKV-Spitzenverband, 2013; KZBV, 2013	Combination of systematic reviews and primary studies	Primary & secondary data	Retention time/length of time remained vital
Schwendicke et al. [4]	CEA	Individual patient-level microsimulation Markov	Mixed public–private-payer	Lifetime	German public and private dental fee catalogues (BEMA and GOZ)	Combination of systematic reviews and primary studies	Primary & secondary data	Tooth retention years
Schwendicke et al. [5]	CEA	Individual patient-level microsimulation Markov	Mixed public–private-payer	Lifetime	German public and private dental fee catalogues (BEMA and GOZ)	Combination of systematic reviews and primary studies	Primary & secondary data	Tooth retention years
Schwendicke et al. [6]	CEA	Not stated	Public-payer perspective (public insurance system)	10 years	German public fee catalogue (BEMA)	Longitudinal and cohort studies	Primary & secondary data	Caries increment; net revenue
Schwendicke et al. [7]	CEA	Not stated	Mixed public–private-payer	Lifetime	German public and private dental fee catalogues (BEMA and GOZ)	Combination of systematic reviews and primary studies	Primary & secondary data	Tooth retention years
Schwendicke et al. [8]	CEA	Individual patient-level microsimulation Markov	Mixed public–private-payer	Lifetime	German public and private dental fee catalogues (BEMA and GOZ)	Combination of systematic reviews and primary studies	Primary & secondary data	Caries increment in DMFT; tooth retention years (secondary)
Splieth et al. [9]	Cost–cost analysis	System dynamics model	Unclear	Lifetime of cohort	German national health system	Effects of fluoride use over time and caries risk (based on local figures); combination of systematic reviews and primary studies	Primary & secondary data	Caries reduction; costs (lifetime, annual)

*CEA* cost effectiveness analysis

All nine studies included in the final analysis were written in the English language. All but one study undertook cost-effectiveness analyses (n = 8, 89%). The remaining study reported a cost-cost analysis (n = 1, 11%). The vast majority of studies were carried out in Germany (n = 8, 89%), with six being carried out by the same first author (66%).

Most studies focused on the prevention or management of dental caries (*n* = 7, 78%), with one study (11%) relating to MIH and one relating to the caries increment as a result of a 20% tax on sugar-sweetened beverages (11%). Just over half of the studies undertook an individual patient-level microsimulation Markov model (n = 5, 56%), one was a cohort simulation Markov model (11%), one system dynamics model (11%) and two did not clearly state their model design (22%). Justification for the choice of model was not always provided. Two thirds of the studies used a lifetime horizon (n = 6, 67%). One study used nine years (11%), one ten years (11%) and whilst the remaining paper used 63.5 years, detailed as the remaining life expectancy of a 15-year old male simulated in their model (11%).

The overall mean percentage of applicable criteria met by the studies in this review for the Drummond Checklist was 82% (median = 85%, range = 54–100%) and for the CHEERS checklist was 82% (median = 87%, range = 32–96%). Applicability scores and an assessment overall quality, based on pre-determined cut-offs [26], of for individual studies are shown in Table 3. In terms of quality, all nine studies were classified as high methodological quality in relation to the Drummond checklist. In comparison, seven studies were categorised as having high reporting quality, one moderate and one low quality in relation to the CHEERS checklist.

**Table 3:** Drummond and CHEERS percentage applicability of each study

Author	% applicable Drummond criteria met	Overall methodological quality	% applicable CHEERS criteria met	Overall reporting quality
Broden et al. [1]	69	High	91	High
Elhennawy et al. [2]	100	High	87	High
Schwendicke et al. [3]	77	High	96	High
Schwendicke et al. [4]	69	High	86	High
Schwendicke et al. [5]	85	High	95	High
Schwendicke et al. [6]	85	High	86	High
Schwendicke et al. [7]	100	High	91	High
Schwendicke et al. [8]	100	High	78	Moderate
Splieth et al. [9]	54	High	32	Low

An overall breakdown of whether studies met each criterion of the Drummond and CHEERS checklists are shown in Tables 4 and 5 respectively. None of the studies reported on the measurement and valuation of preference-based outcomes, however, such outcome measures are more commonly applicable to cost-utility or cost–benefit analyses, of which there were none identified in this review.

**Table 4:** Breakdown of studies meeting each Drummond Criterion

Drummond criterion	Summary of criterion	Total studies meeting criterion n = 9	Total studies not meeting criterion n = 9	Total studies where criterion was not applicable n = 9
1	Was a well-defined question posed in answerable form?	9	0	0
2	Was a comprehensive description of the competing alternatives given?	6	3	0
3	Was there evidence that the programme’s effectiveness had been established?	7	2	0
4	Were all the important and relevant outcomes and costs for each alternative identified?	6	3	0
5a	Were outcomes measured accurately in appropriate units prior to evaluation?	9	0	0
5b	Were costs measured accurately in appropriate units prior to evaluation?	9	0	0
6a	Were the outcomes valued credibly?	9	0	0
6b	Were the costs valued credibly?	9	0	0
7a	Were outcomes adjusted for different times at which they occurred?	9	0	0
7b	Were costs adjusted for different times at which they occurred?	3	6	0
8	Was an incremental analysis of the outcomes and costs of alternatives performed?	5	4	0
9	Was allowance made for uncertainty in the estimates of costs and consequences?	8	1	0
10	Did the presentation and discussion of study results include all of the issues that are of concern to users?	8	1	0

**Table 5:** Breakdown of studies meeting each CHEERS Criterion

CHEERS Criterion	Summary of criterion	Total studies meeting criterion n = 9	Total studies not meeting criterion n = 9	Total studies where criterion was not applicable n = 9
1	Title	7	2	0
2	Abstract	9	0	0
3	Background and objectives	9	0	0
4	Target population and subgroups	7	2	0
5	Setting and Location	9	0	0
6	Study perspective	9	0	0
7	Comparators	8	1	0
8	Time horizon	9	0	0
9	Discount rate	8	1	0
10	Choice of health outcomes	8	1	0
11^a^	Measurement of effectiveness	8	1	0
12	Measurement and valuation of preference-based outcomes	0	0	9
13^a^	Estimating resources and costs	8	1	0
14	Currency, price date and conversion	6	2	1
15	Choice of model	4	5	0
16	Assumptions	8	0	1
17	Analytical methods	4	5	0
18	Study parameters	8	1	0
19	Incremental costs and outcomes	7	2	0
20^a^	Characterising uncertainty	7	2	0
21	Characterising heterogeneity	3	1	5
22	Study findings, limitations, generalisability and current knowledge	9	0	0
23	Source of funding	4	5	0
24	Conflicts of interest	7	2	0

^a^Model-based criterion used

## Discussion

To the author’s knowledge, this is the first systematic review that has assessed the methodological and reporting quality of DAM studies, with horizons extending beyond childhood, within the field of child oral health. Limited examples of using DAMs exist in the field of child oral health; however, those that do exist are of relatively high methodological and reporting quality.

Using the high-, moderate- and low-level cut-offs of quality, as defined by Rogers et al. [26], the overall methodological quality was deemed to be high for all included studies in this review. In their study, Rogers et al. [26] reported that only 50% (n = 23) of included studies were classified as high methodological quality. The median score for overall methodological quality in this review was 85%, meaning that on average studies met 11 out of the 13 criterion of the Drummond Checklist [27]. This was much higher than the median score of 50% reported by Rogers et al. [26] It is unlikely that the threshold for the studies included in the Rogers et al. [26] review (studies were excluded if they involved decision models that extended into adulthood, or over a lifetime as to focus on the benefits from interventions gained solely during the childhood period) was the reason behind these differences. One possible explanation could be that the studies included in Rogers et al. [26] review were, in general, slightly older studies and therefore pre-date the Drummond checklist, which now commonly acts as a guide and an evaluation tool for economic evaluations. Alternatively, it could be that undertaking a decision analytical model that extends into adulthood, or over a lifetime, is methodologically more complex [19], and therefore in order to undertake the analysis many of the criteria listed in the Drummond checklist [27] were met as they were required to execute the model. Or, the number of authors to included studies ratio in each review may explain these differences. Rogers et al. [26] included a total of 46 studies led by 43 different authors. Whereas, of the nine studies included in this review, seven were carried out by the same German research group. It should be noted that the outcome of these DAMs is only pertinent to a German population, as the chosen perspective was that of a German mixed public–private-payer, and thus generalising these results outside of Germany is not permissible. However, this group’s positive adherence to the guidance [31] and Drummond checklist [27], as shown by their high applicability of criterion met, could suggest why a high overall median value of methodological quality was obtained for this review. Interestingly, the median value for this review was consistent with that reported by Tonmykayakul et al. [23] for full economic evaluations in the wider field of dentistry, although, they did not specify which of the economic evaluations ran in conjunction with a randomised control trial, or indeed included a decision analytical model, and therefore a meaningful comparison cannot be made.

Discounting of costs and performing an incremental analysis of the costs and outcomes were the most omitted criterion from studies included in this review. Future costs need to be discounted to reflect the amount spent or saved in the future should not weigh as heavily in decisions as those spent or saved today [19, 32]. This criterion is important for modelling studies in oral health interventions in children as dental disease is often a ‘chronic’ issue and the costs and outcomes associated with each decision will extend well into adulthood. The longer the horizon of the model, the more significant the difference is noted if discounting is not undertaken [19]. Therefore, as most studies included in this review used a lifetime horizon, by not discounting the costs could significantly impact the result.

Another criterion that was frequently absent was an incremental analysis of the outcomes and costs of alternatives. Omitting this analysis fails to provide the stakeholders, for whom the results from any modelling study will be essential to guide clinical and policy decisions, as to whether the additional costs generated by one alternative over another is compared to the additional benefits generated. It could be argued that an incremental analysis is a fundamental output of any given economic evaluation [16].

The overall reporting quality of economic evaluations included in this review was relatively high, with seven out of nine studies being in the high category. A median score of 87% against the CHEERS checklist was noted, which was similar to the median score of 83% reported by Rogers et al. [26]. Interestingly, the only study that was classified as low in this review was the oldest study, and similar observations were observed in the other review [26]. Reporting of economic evaluations of health interventions has been known to pose challenges, with various journals in the past offering their own advice [29]. The development of the CHEERS checklist in 2013 attempted to consolidate and update the various existing guidance into one useful resource [29]. This could explain why in both reviews the more recently published studies were noted to have a much higher reporting quality.

The worst performing criteria, in relation to the CHEERS checklist, were reporting the choice and justification of the chosen model, analytical methods used and sources of funding. Describing, but more importantly, justifying the choice of model, for example Markov-model or decision tree, is an essential piece of information needed for any modelling study [33]. The type of model chosen should be driven by the clinical problem, with the model’s structure being sufficiently complex to answer the proposed question in enough detail to ensure adequate external validity [31]. Without such information, the reader is unable to ascertain whether this is the best model to adequately address the decision problem in the given clinical or policy context. Similarly, a clear description of all analytical methods, including how to deal with uncertainties incorporated into the model, should be provided to ensure the results are valid [34]. Despite four studies failing to meet this criterion in this review, they did all provide some form of detail relating to their analytical approach. However, for a study to have met this criterion, for the purposes of this review, it was agreed between the reviewers that at least three of the five subsections had to have been reported. Finally, several studies failed to report their sources of funding, even if none existed. This declaration is important as non-disclosure could lead to a funding bias, and selective reporting of results [35].

The majority of the modelling studies in this review focussed on the prevention and/or management of dental caries. Similarly, Rogers et al. [26] noted that 42 (91%) of the studies included in their review was to deal with the prevention and/or management of caries. This is understandable given the prevalence of this dental condition amongst children [3]. Adopting a modelling approach, rather than a trial-based economic evaluation, is sensible for the management of dental caries in the permanent dentition as a randomised control trial is unlikely to determine the life-long impact on patients given the usual short-term follow-up of a trial. However, modelling approaches are not as important for management of dental caries in the primary dentition given their natural exfoliation occurs in childhood. A recent systematic review by Qu et al. [24] reported the application of DAM, and their methodological quality, in the context of dental caries across all age ranges. They included 25 studies and demonstrated that a variety of model structures were used, with a Markov model being most common [24]. They reported that the methodological quality of DAM in dental caries-related economic evaluations were unsatisfactory [24]. However the methodology of the review is unclear as some of the outcome measures listed in the included studies were not identified in the inclusion criteria.

Only one study in this review was not related to dental caries. Other dental conditions observed in children would benefit from modelling studies, for example management options for non-vital teeth with immature apices, management of anterior opacities due to MIH or the management of traumatic dental injuries. Although these conditions may not be as prevalent, these are just as impactful, and therefore this evidence found in this review supports the argument set out in Rogers et al. [26] that other conditions relating to child oral health should be priorities for future economic evaluation research.

All but one of the modelling studies included in this review conducted a cost-effectiveness analysis. Most of the studies either used tooth retention years or caries increment as their outcome measure, which are appropriate when being applied to modelling studies, as they are both able to capture long-term outcomes associated with the interventions. This predominance of cost-effectiveness analyses is consistent with previous systematic reviews addressing oral health interventions [23, 26]. However other approaches to economic evaluation exist and should be considered in the field of child oral health. In the UK, the National Institute for Health Care and Excellence recommend cost-utility analyses, with results being reported in terms of the incremental cost per quality adjusted life year (QALY) gained [36]. Thus, allowing comparison across different areas of healthcare using a measure of health benefit that combines both quality of life and length of life into a single index [36]. Hettiarachchi et al. [25] did report that an increasing trend in the publication of cost-utility analyses in dentistry, however this was not observed in the present review as no modelling studies adopted a cost-utility analytical approach. This is not surprising, as commonly used tools to derive QALYs in children, have not been applied to oral health conditions other than caries. Foster-Page et al. [37] found that one tool, the CHU9D, was not sensitive enough to detect changes in caries status. However, it is worth noting that oral health specific instruments are emerging [38, 39]. Alternatively, a cost–benefit analysis could be undertaken, where commensurate units (usually money) are used to value both costs and outcomes. Establishing willingness-to-pay values from preference elicitation methods, such as discrete choice experiments, can be used as a proxy for utility and therefore be used as an outcome measurement. These monetary values can be attached to health states in a model, in a similar way the units of outcomes for cost-effectiveness and cost-utility models are. This approach could enable comparison across various dental disease profiles, which cannot always occur in cost-effectiveness analysis as the diseases studied may be measured in different units.

## Strengths and limitations

This is the first systematic review that has addressed the scope and quality of economic evaluations that involve decision analytical models extending into adulthood in the field of child oral health. A strength of this review was the involvement of both paediatric dental and health economics expertise at each stage of the review. Furthermore, a calibration exercise was undertaken for data extraction and quality assessment, against both checklists, which was beneficial as shown by the intra- and inter-rater agreements scores. In addition, this review complemented the recent review by Rogers et al. [26], by overcoming their main limitation of excluding modelling studies which included decisions made about young people over the age of 18-years.

One acknowledged limitation was adopting the same cut-offs that were defined by Rogers et al. [26] These cut-offs were ascertained by using the median percentage, and interquartile range, of Drummond and CHEERS criteria met by the studies in their review, in an attempt to ensure that studies with a greater number of ‘not applicable’ criteria were not unfairly disadvantaged [26]. It could be argued that this review should have adopted a similar methodology, although, it was agreed by the research team that adopting these same cut-offs for high-, moderate- and low-quality studies would allow for a meaningful and direct comparison to a very similar and recent systematic review. The percentage for those studies classified as high in this review were all significantly higher than the predetermined cut-offs used. Incidentally, the number of criterion ‘not applicable’ to studies in this review was much less, comparatively, to that of Rogers et al. [26], and had cut-offs been created specifically for this review, the values would likely have all been higher. Nonetheless, this would have made very little difference to the overall quality assessment given the high percentages observed for each study, and each evaluation checklist, in this review.

## Conclusion

There is a paucity of decision analytical models in the field of child oral health which span beyond the age of 18, however the few that are available are of relatively high methodological and reporting quality. Decision analytical models can help, and should be used to, understand the implications of dental decisions made in children whereby the outcomes extend beyond 18 years old, and further work using this methodology in child oral health would be valuable.

## Supplementary Information

**Additional file 1.** Search Strategy.

## Data Availability

All data generated or analysed during this study are included in this published article and its Additional file 1.

## Abbreviations

CHEERS: Consolidated Health Economic Evaluation Reporting Standards
DAM: Decision analytic models
MIH: Molar incisor hypomineralisation
PRISMA: Preferred Reporting Items for Systematic Reviews and Meta-Analysis
QALY: Quality adjusted life year
