# Supplementary material for: A systematic review of the quality and scope of decision modelling studies in child oral health research
Source: BMC Oral Health. 2021 Jun 25;21:318. doi: 10.1186/s12903-021-01680-3 (PMC8229274; doi:10.1186/s12903-021-01680-3)
Supplement: Supplementary file 1 — Additional file 1. Search Strategy. [file 12903_2021_1680_MOESM1_ESM.docx]

**Title:** A systematic review of the quality and scope of decision modelling studies in child oral health research

**Authors:**

Mr Greig D.Taylor^1,2^, Miss Katherine Carr^1^, Miss Helen J Rogers^3^ and Dr Chris R Vernazza^1,2^

**Supplementary File: Search Strategy**

| 1. "Dental Care for Children"/ec [economics] |
| --- |
| 2. Health Education, Dental/ec [economics] |
| 3. Oral health/ec [economics] |
| 4. (Dent* or Teeth or "oral health" or "oral surgery" or orthodont* or "fluoride" or "hypomineralisation" or "hypomineralization" or "caries").ti. |
| 5. 1 or 2 or 4 |
| 6. Child/ |
| 7. Adolescent/ or Infant/ |
| 8. (child* or p?ediatric* or infan* or adolescen* or teen*).ti. |
| 9. 6 or 7 or 8 |
| 10. 5 and 9 |
| 11. Economics/ |
| 12. exp "costs and cost analysis"/ |
| 13. Economics, Dental/ |
| 14. exp economics, hospital/ |
| 15. (economic$ or cost or costs or costly or costing or price or prices or pricing).ti. |
| 16. (expenditure$ not energy).ti. |
| 17. "value for money".ti. |
| 18. budget$.ti. |
| 19. or/11-18 |
| 20. ((energy or oxygen) adj cost).ti,ab. |
| 21. (metabolic adj cost).ti,ab. |
| 22. ((energy or oxygen) adj expenditure).ti,ab. |
| 23. or/20-22 |
| 24. 19 not 23 |
| 25. letter.pt. |
| 26. editorial.pt. |
| 27. historical article.pt. |
| 28. or/25-27 |
| 29. 24 not 28 |
| 30. exp animals/ not humans/ |
| 31. 29 not 30 |
| 32. bmj.jn. |
| 33. "cochrane database of systematic reviews".jn. |
| 34. health technology assessment winchester england.jn. |
| 35. or/32-34 |
| 36. 31 not 35 |
| 37. 10 and 36 |
| 38. (model or models or modelling or markov or decision or analytic or lifetime or horizon).mp. [mp=tx, bt, ti, ab, ct, hw, ar, ax, bx, cx, ft, fd, ad, jh, jx, ca, pc, ot, nm, fx, kf, ox, px, rx, ui, sy, tc, id, tm, mh] |
| 39. 37 and 38 |
| 40. (socioeconomic or socio-economic or ecology or ecological).ti. |
| 41. 39 not 40 |
